# DC-SIGN on B Lymphocytes Is Required For Transmission of HIV-1 to T Lymphocytes

**DOI:** 10.1371/journal.ppat.0020070

**Published:** 2006-07-14

**Authors:** Giovanna Rappocciolo, Paolo Piazza, Craig L Fuller, Todd A Reinhart, Simon C Watkins, David T Rowe, Mariel Jais, Phalguni Gupta, Charles R Rinaldo

**Affiliations:** 1 Department of Infectious Diseases and Microbiology, Graduate School of Public Health, University of Pittsburgh, Pittsburgh, Pennsylvania, United States of America; 2 Department of Cell Biology and Physiology, School of Medicine, University of Pittsburgh, Pittsburgh, Pennsylvania, United States of America; 3 Department of Pathology, School of Medicine, University of Pittsburgh, Pittsburgh, Pennsylvania, United States of America; National Institutes of Health-NIAID, United States of America

## Abstract

Infection of T cells by HIV-1 can occur through binding of virus to dendritic cell (DC)-specific ICAM-3 grabbing nonintegrin (DC-SIGN) on dendritic cells and transfer of virus to CD4^+^ T cells. Here we show that a subset of B cells in the blood and tonsils of normal donors expressed DC-SIGN, and that this increased after stimulation in vitro with interleukin 4 and CD40 ligand, with enhanced expression of activation and co-stimulatory molecules CD23, CD58, CD80, and CD86, and CD22. The activated B cells captured and internalized X4 and R5 tropic strains of HIV-1, and mediated *trans* infection of T cells. Pretreatment of the B cells with anti–DC-SIGN monoclonal antibody blocked *trans* infection of T cells by both strains of HIV-1. These results indicate that DC-SIGN serves as a portal on B cells for HIV-1 infection of T cells in *trans*. Transmission of HIV-1 from B cells to T cells through this DC-SIGN pathway could be important in the pathogenesis of HIV-1 infection.

## Synopsis

A cell surface molecule, DC-SIGN, is known to bind the AIDS virus, human immunodeficiency virus 1 (HIV-1), on dendritic cells. HIV-1 can then be transferred from these dendritic cells to CD4^+^ T cells, in which the virus replicates and kills the T cells. Here, Rappocciolo and colleagues present their findings that DC-SIGN serves a similar function on a subset of B cells of the peripheral blood and tonsils. Although B cells that express DC-SIGN do not replicate HIV-1, they serve as portals for transfer and enhanced HIV-1 infection of CD4^+^ T cells, the major site of virus replication in the host. This newly described pathway for HIV-1 infection of T cells via B cells could be important in the pathogenesis of the virus infection.

## Introduction

HIV-1 can bind to the type II C-type lectin receptor, dendritic cell (DC)-specific ICAM-3 grabbing nonintegrin (DC-SIGN; CD209), on myeloid DC and be transferred to CD4^+^ T cells [1,2]. An important feature of this *trans* pathway is that the virus does not establish an efficient, productive infection in these DC. Rather, it is captured by the DC and internalized in distinct intracellular compartments, and then transmitted to CD4^+^ T cells wherein it undergoes productive replication [3]. This is considered to be an alternative pathway to HIV-1 *cis* infection of T cells, macrophages, and DC that occurs through binding to the primary CD4 receptor and either of the chemokine coreceptors CXCR4 or CCR5.

B lymphocytes have also been implicated in *trans* infection of T cells with HIV-1 [4]. Given the intimate association of B cells and T cells in lymphoid tissue, B cell–mediated *trans* infection pathways could be important in the spread of virus to T cells. B cells derived either from lymphoid tissue or from the peripheral blood of HIV-1–infected persons carry replication-competent virus of either the CXCR4 (X4) or CCR5 (R5) tropic strain [5]. The mechanism by which B cells have been shown to transmit the virus involves binding of HIV-1 immune complexes to CR2 or CD21 on the surface of B cells and subsequent passage to the T cells [6–10]. Other reports have proposed a role for B cells in HIV-1 infection involving B cell activation processes induced by *nef*-expressing macrophages [11].

B cells express some C-type lectin receptors [12–15], with conflicting reports on expression of DC-SIGN [16,17]. Transfer of HIV-1 by B cells via a C-type lectin receptor pathway described for DC could be of significance in HIV-1 pathogenesis. We therefore investigated B cells for a DC-SIGN–mediated, *trans* pathway for HIV-1 infection of CD4^+^ T cells. We found that a subset of B cells in the peripheral blood and tonsils of healthy, HIV-1 seronegative donors expressed DC-SIGN, and that DC-SIGN expression increased after stimulation with interleukin 4 (IL-4) and CD40 ligand (CD40L). The stimulated, DC-SIGN^+^ B cells mediated *trans* infection of T cells.

## Results

### DC-SIGN Expression in B cells and Enhancement by Stimulation with IL-4 and CD40L

**Figure 1:**
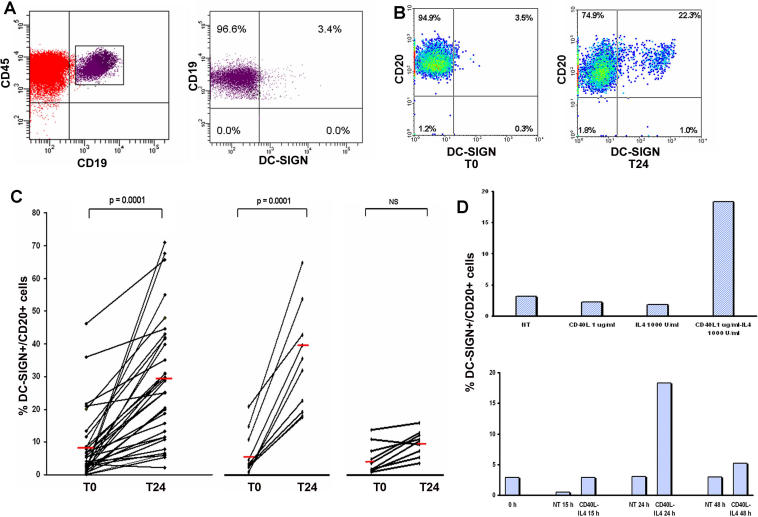
Expression of DC-SIGN on B Cells (A) B cells within the PBMC population expressed DC-SIGN. PBMC were assessed for expression of CD45, CD19, and DC-SIGN by flow cytometry. Cells coexpressing CD45 and CD19 (left) were analyzed for DC-SIGN coexpression (right). Quadrant positions were determined with the isotype controls. (B) Increase in coexpression of DC-SIGN on CD20^+^ cells detected by flow cytometry in unstimulated, purified, fresh B cells (0 h; T0) and IL-4– and CD40L-stimulated, purified B cells at 24 h (T24). Data are from a representative, healthy HIV-1–seronegative donor. (C) DC-SIGN expression on B cells from healthy, HIV-1–seronegative donors (*n* = 33, left), HIV-1–seropositive, ART therapy–naive subjects (*n* = 10, middle), and HIV-1–seropositive subjects receiving ART (*n* = 10, right). Quadrants were positioned on the isotype controls. (D) CD40L and IL-4 acted synergistically in inducing enhanced DC-SIGN expression in B cells by 24 h. In this representative experiment (top), purified B cells were cultured in the presence of IL-4 or CD40L or a combination of the two. Untreated B cells were used as controls (NT). DC-SIGN and CD20 coexpression on activated B cells was greatest using a combination of 1,000 U/ml of IL4 and 1 μg/ml of CD40L for 24 h (bottom). Single concentrations of 100, 500, and 2,000 U/ml of IL-4 and 0.1, 0.5, and 10 μg/ml of CD40L induced similar, low levels of DC-SIGN expression (unpublished data); various combinations of these concentrations of IL-4 and CD40L induced less DC-SIGN expression than this combination (unpublished data).

Our initial, three-color flow cytometric analysis of peripheral blood mononuclear cells (PBMC) showed that DC-SIGN was expressed on a small but distinct subset of CD19^+^ B cells within the CD45^+^/CD19^+^ gated population of PBMC of normal, HIV-1–negative persons (representative example, Figure 1A). To extend this finding, we analyzed DC-SIGN expression on B cells that were purified from PBMC of 33 normal donors by sorting with anti-CD19 monoclonal antibody (mAb)-coated magnetic beads. Flow cytometric results showed that DC-SIGN was expressed on 7.9 ± 1.8% (mean ± standard error [SE]) of purified, peripheral blood CD20^+^ B cells (representative example, Figure 1B, T0; cumulative results, Figure 1C, T0).

We next addressed whether expression of DC-SIGN on this subset of B cells was related to a state of cellular activation. For this, we determined the proportion of B cells that expressed CD23, another type II C-type lectin receptor that is a low-affinity Fc receptor for IgE (Fc epsilon RII) and is associated with B cell activation and differentiation [15]. We found that DC-SIGN and CD23 were coexpressed on 8.1 ± 1.7% of B cells in normal persons (Figure S1A, T0). Indeed, 80% of DC-SIGN^+^ B cells also expressed CD23 (unpublished data). To further delineate the relationship of B cell activation to DC-SIGN, we examined whether stimulation of B cells with the T helper type 2 (Th2) cytokine interleukin 4 (IL-4) alone or in combination with the CD4^+^ T cell activation factor CD40L could alter expression of DC-SIGN and CD23, a procedure that mimics activation of B cells by CD4^+^ T helper cells during antigen processing [18,19]. Also, IL-4 has been shown to up-regulate expression of DC-SIGN on monocyte-derived DC [20], breast-milk macrophages [21], and the THP-1 cell line [22]. Our data demonstrate that treatment of purified B cells with a range of concentrations of either IL-4 or CD40L alone had little effect on the expression of DC-SIGN or CD23 by 24 h (Figure 1D, top; Figure S1B, top) or 48 h (unpublished data). In contrast, treatment with a combination of different concentrations of IL-4 and CD40L had a synergistic effect on expression of DC-SIGN and CD23 on B cells, with the greatest increase in the number of B cells expressing DC-SIGN being induced by stimulation with 1,000 U/ml of IL-4 and 1 μg/ml of CD40L for 24 h (Figure 1D, top and bottom, respectively; Figure S1B, bottom). This treatment was therefore used for stimulation of B cells in subsequent experiments.

Cumulative data from 33 normal donors showed that expression of DC-SIGN by purified, peripheral blood CD20^+^ B cells significantly increased after stimulation of the cells for 24 h with IL-4 and CD40L (representative example, Figure 1B) from a mean level of 7.9 ± 1.8% to 28.2 ± 3.3% (*p* = 0.0001) (Figure 1C). Moreover, the mean fluorescence intensity (MFI) of DC-SIGN on the B cells increased 3-fold after stimulation, from 10.8 ± 2.4 to 30.2 ± 6.6 (*p* < 0.005). The DC-SIGN^+^ CD23^+^ B cell population also increased from 8.1 ± 1.7% at 0 h to 25.7 ± 3.2% at 24 h (*n* = 33; *p* < 0.0002; Figure S1A, T24), with 86% of DC-SIGN^+^ cells expressing CD23 after 24 h of stimulation (unpublished data).

To confirm the increase in DC-SIGN expression in B lymphocytes stimulated by IL-4 and CD40L, we measured the level of DC-SIGN mRNA by real-time reverse transcriptase PCR [23,24] in B cells stimulated with IL-4 and CD40L compared to unstimulated B cells. B cells from five of seven normal donors (donors 1, 2, 4, 5, and 7) had large, 6.1- to 122.4-fold increases in levels of DC-SIGN mRNA, accompanied by an increase in surface expression of DC-SIGN (Table 1). B lymphocytes from the other two donors (donors 3 and 6) had low-to-moderate, 1.1- to 2.1-fold increases in DC-SIGN mRNA levels together with increases in surface expression of DC-SIGN after stimulation.

**Table 1:**
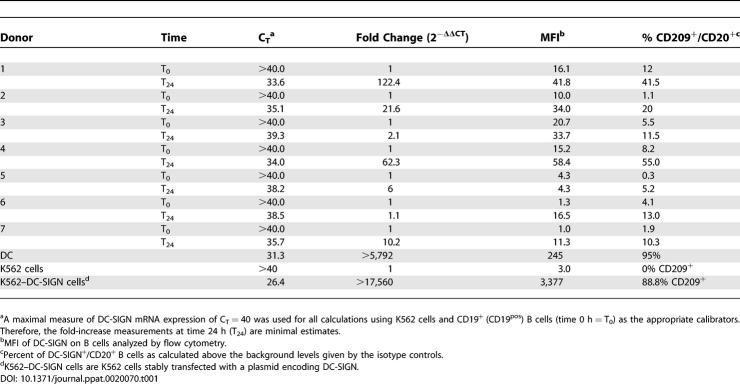
Real-Time RT-PCR Measurement of DC-SIGN mRNA Levels in B cells of Seven Healthy, HIV-1 Seronegative Donors

We next examined whether HIV-1 infection altered the number of B cells expressing DC-SIGN in the peripheral blood of HIV-1–infected persons, and the capacity of their B cells to respond to stimulation with IL-4 and CD40L. We found that the percentage of DC-SIGN^+^ B cells in the blood of HIV-1–infected persons with chronic HIV-1 infection who were antiretroviral therapy (ART) naive or those who had suppressed viral infection on ART was similar to uninfected persons (Figure 1C). However, expression of DC-SIGN was not enhanced on B cells from the HIV-1–infected subjects on ART in response to stimulation with IL-4 and CD40L, whereas DC-SIGN expression was enhanced in B cells from HIV-1–uninfected persons and ART-naive, HIV-1–infected subjects.

**Figure 2:**
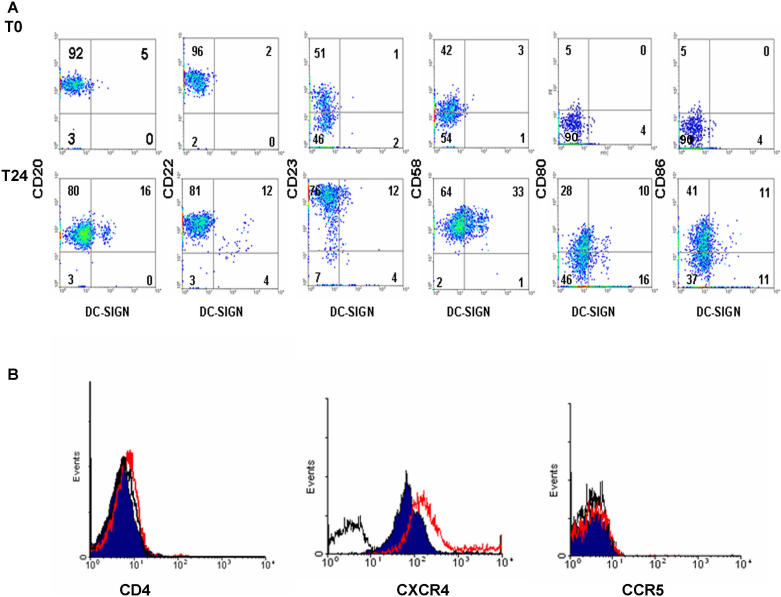
Coexpression of DC-SIGN and Markers of Activation on B Cells Expression of CD20, CD22, CD23, CD58, CD80, and CD86 in purified, unstimulated ([A] time 0 h; T0) and IL-4– and CD40L-stimulated ([B] time 24 h; T24) B cells. The quadrants were set on the isotype controls. Expression of CD4, CXCR4, and CCR5 is shown for unstimulated (full histograms) and stimulated (24 h; red profile histograms), purified B lymphocytes. Isotype controls are indicated by gray lines. Data are from a single experiment representative of five independent experiments.

Next we compared the level of expression of DC-SIGN to other surface molecules that are known to increase during B cell activation and that play an important role in the interaction between B and T lymphocytes. Flow cytometry analysis of purified, IL-4– and CD40L-stimulated B cells for expression of DC-SIGN, CD23, the B cell signal transduction molecule CD22, and T cell co-stimulatory molecules CD58, CD80, and CD86 showed that there was an increase in coexpression of all of these markers with DC-SIGN at 24 h compared to 0 h (*p* < 0.005; T0 and T24, Figure 2A) or mock-stimulated B cells at 24 h (*p* < 0.05; unpublished data).

Finally, we determined the expression of CD4 and the chemokine receptors CXCR4 and CCR5 on B cells, which are the primary receptor and coreceptors involved in HIV-1 *cis* infection. Although a small subset of B cells from the blood expressed low levels of CD4, this marker was not detectable after 24 h (Figure 2B) or 48 h (unpublished data) of stimulation of the B cells with IL-4 and CD40L. High levels of expression of the CXCR4 coreceptor for HIV-1 were evident on most B cells before and after stimulation with IL-4 and CD40L, whereas the CCR5 coreceptor was not expressed.

Taken together, these results indicate that a distinct population of activated B lymphocytes constitutively expressed DC-SIGN in the blood of normal donors. Moreover, there was a significant increase in the frequency and intensity of DC-SIGN–expressing B cells derived from HIV-1–negative subjects and HIV-1–infected persons not on ART, which was not observed in HIV-1–infected persons on ART, after 24 h of stimulation with IL-4 and CD40L. Finally, the activated B cells expressed the CXCR4 coreceptor for HIV-1, but not the primary CD4 receptor or the CCR5 coreceptor for the virus.

### B Cells Express Sufficient Levels of DC-SIGN for HIV-1 *trans* Infection of T Cells

**Figure 3:**
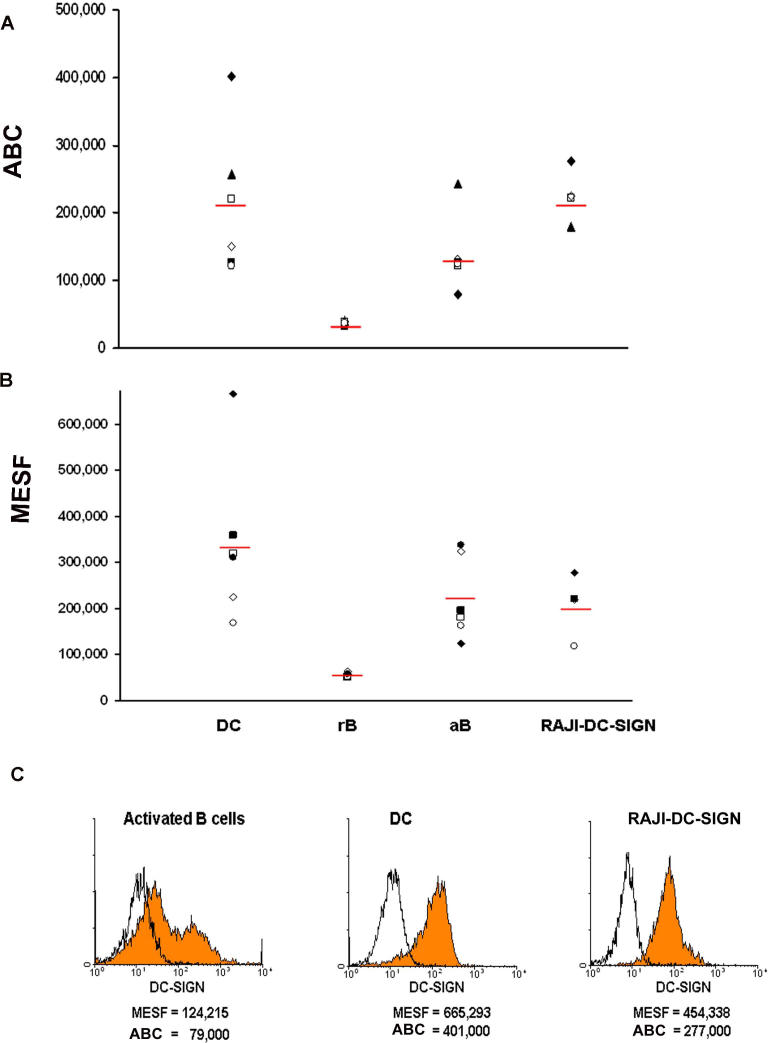
Quantitation of DC-SIGN Expression on B Cells ABC and MESF (panels [A] and [B], respectively) of unstimulated (resting [rB]) and activated (aB) B cells and DC from six donors, and four independent samplings of Raji–DC-SIGN cells, were determined as described in Materials and Methods. *p* < 0.008 for ABC and *p* < 0.001 for MESF comparing rB to aB cells; *p* = NS comparing aB cells to DC or Raji–DC-SIGN cells. The red lines indicate the mean values for ABC and MESF. In panel (C), the histogram profiles of expression of DC-SIGN on activated B cells and DC from one representative donor are shown, as well as the profile of expression of DC-SIGN on Raji–DC-SIGN cells for comparison (full histograms: DC-SIGN positive cells, black line overlay histograms: isotype control).

It has been shown in both DC and transfected cell lines that surface expression of a minimum of approximately 60,000 molecules of DC-SIGN is necessary for transmission of HIV-1 to T cells [25]. We therefore determined the level of expression of DC-SIGN on blood-derived B cells as compared to DC and Raji–DC-SIGN cells by antibody binding capacity (ABC) and the number of molecules of equivalent soluble fluorochrome (MESF). The results show that IL-4– and CD40L-stimulated B cells expressed 137,870 ± 22,432 ABC (*n* = 6) for DC-SIGN compared to mock-stimulated B cells (36,946 ± 1,125, *n* = 6), DC (213,350 ± 43,370 ABC; *n* = 6), and Raji–DC-SIGN cells (225,750 ± 19,880 ABC; *n* = 4) (Figure 3A). A similar quantitative expression of DC-SIGN was observed on activated B cells by MESF (Figure 3B). The MESF and ABC histogram profiles for DC-SIGN on activated B cells compared to DC and Raji–DC-SIGN cells are shown in Figure 3C. These data support that IL-4– and CD40L-stimulated B cells expressed a sufficient number of DC-SIGN molecules for transfer of HIV-1 to T cells.

### Activated B Cells Transmit HIV-1 X4 and R5 Strains to T Lymphocytes

**Figure 4:**
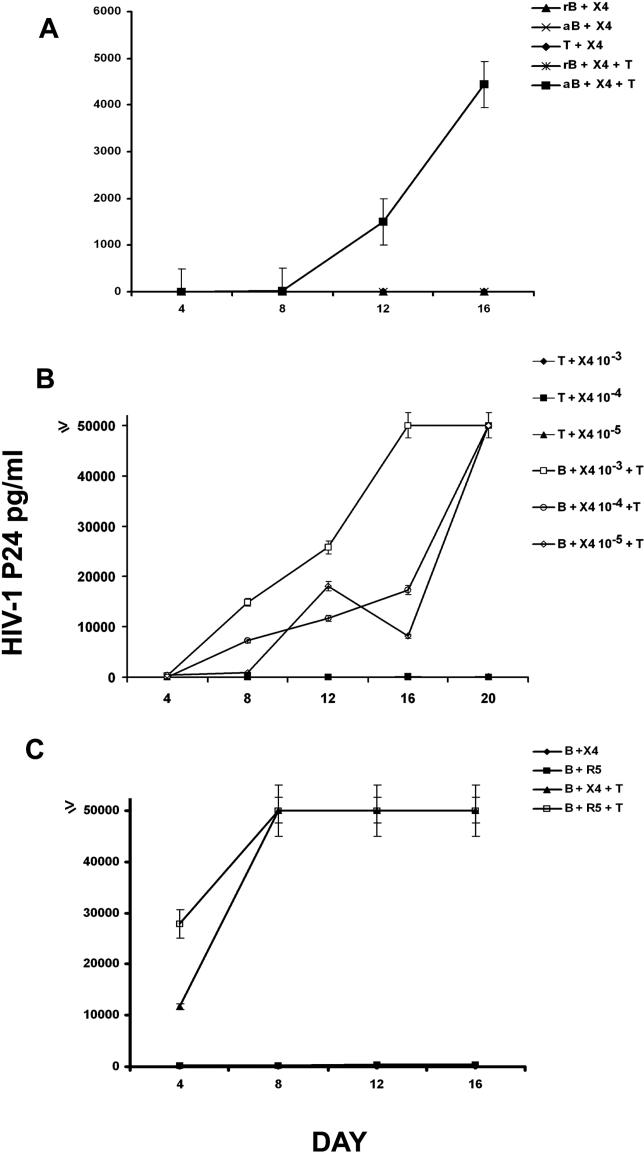
Activated B Cells Transmit HIV-1 X4 and R5 to T Cells (A) Levels of HIV-1 p24 in B cells stimulated with IL-4 and CD40L (activated B cells [aB]) or mock stimulated (resting B cells [rB]) for 24 h and loaded with 10^−4^ MOI of HIV-1 X4 (IIIb) for 2 h at 37 °C, then extensively washed in cold medium and incubated with purified, autologous CD4^+^ T cells (T). B cells and T cells directly loaded with HIV-1 served as controls. (B) Dose response of HIV-1 *trans* infection shown by levels of p24 in cultures of autologous T cells (T) mixed with activated B cells (B) that were loaded with 10^−5^, 10^−4^, or 10^−3^ MOI of HIV-1 X4 (IIIb). The results shown are representative of three independent experiments with B and T cells from different donors. (C) Activated B cells (B) transmitted both HIV-1 X4 (IIIb) and R5 (Ba-L) strains to autologous T cells (T) as shown by increases in levels of p24. T cells and B cells were separately cultured with either strain of virus as controls. Data are represented as the means of triplicates ± SE, and are from single experiments representative of five or more independent experiments.

Our finding that B lymphocytes expressed DC-SIGN led us to investigate whether these cells could be exploited by HIV-1 as a means for enhanced infection of T cells, as has been demonstrated for DC [1,26]. We first examined whether HIV-1 was able to bind to B cells and be transmitted to T lymphocytes through DC-SIGN using a low concentration of HIV-1 (multiplicity of infection [MOI] = 10^−4^, corresponding to 10 pg of p24 per 10^6^ cells), similar to that used in studies of DC-SIGN–related transmission of HIV-1 from DC to T cells [1,27]. This low amount of virus usually does not result in efficient *cis* infection of T cells. As displayed in Figure 4A, when the purified IL-4– and CD40L-stimulated B cells were incubated with X4 tropic HIV-1 (strain IIIb) and then co-cultured with autologous CD4^+^ T cells, virus replicated in the cultures as shown by an increase from undetectable, <1 × 10^1^ pg of p24 per ml at day 4, to >4 × 10^3^ pg at day 16. Purified B cells not stimulated with IL-4 and CD40L, and loaded with HIV-1 X4 and cultured with T cells for 24 h, were not capable of enhancing HIV-1 infection in the co-cultures. Levels of HIV-1 X4 remained below detection in IL-4– and CD40L-stimulated B cells, mock-stimulated B cells, and T cells alone (Figure 4A). This indicates that virus replication in the B–T cell co-cultures was not a result of direct, *cis* infection of the T cells or B cells by HIV-1. For these HIV-1 transmission experiments, we used B cells obtained from magnetic bead–purified fractions with >96% CD20^+^ cells and <1% T cells and monocytes. However, to ensure that the observed results were not due to contamination with other cell types, we also found that fractions of >96% pure B cells obtained by flow cytometry sorting were able to transmit HIV-1 to T cells with the same efficiency as the magnetic bead–purified B cells (unpublished data).

Further experiments demonstrated that IL-4– and CD40L-activated B cells loaded with three different concentrations of HIV-1 X4 resulted in a dose response–related level of infection in the B–T cell co-cultures (Figure 4B). Levels of HIV-1 p24 increased from undetectable, <1 × 10^1^ pg per ml on day 4, to ≥5 × 10^4^ pg of p24 per ml on day 16 at the highest input MOI (i.e., 10^−3^), and >1.7 × 10^4^ and >8 × 10^3^ pg per ml at the lower, 10^−4^ and 10^−5^ MOI, respectively. Virus did not replicate at any of these input concentrations in B cells or T cells alone (Figure 4B).

We next investigated whether expression of CD4, CXCR4, or CCR5 by B cells was related to *trans* infection of T cells. As shown in Figure 2B and in the literature [28], low numbers of unstimulated B cells in blood express very low levels of CD4. Moreover, expression of CD4 was down-regulated after stimulation of the B cells with IL-4 and CD40L for 24 h (Figure 2B). We next demonstrated that activated B cells enhanced *trans* infection of CD4^+^ T cells with a low MOI of the HIV-1 R5 tropic, Ba-L strain (Figure 4C), comparable to HIV-1 X4, even though B cells do not express CCR5 (Figure 2B) [28]. Moreover, *cis* infection of either unstimulated B cells (unpublished data), stimulated B cells (Figure 4C), or T cells (Figure 4C) with HIV-1 R5 at this low MOI did not result in virus replication.

Taken together, our data indicate that IL-4– and CD40L-activated B cells from HIV-1–negative persons mediated efficient *trans* infection of autologous CD4^+^ T cells with either X4 or R5 tropic HIV-1. Furthermore, this *trans* infection was not related to CD4, CXCR4, or CCR5 expression on the B cells. The fact that these were purified B cells from HIV-1–negative persons indicates that HIV-1 immune complexes and other types of cells were not involved in this process. It should also be noted that we did not include the lectin polybrene, a commonly used, receptor-independent enhancer of HIV-1 infection [29], in any of the cultures.

### B Cell–Mediated Transmission of HIV-1 to T Cells Is Dependent on Expression of DC-SIGN by the B Cells

**Figure 5:**
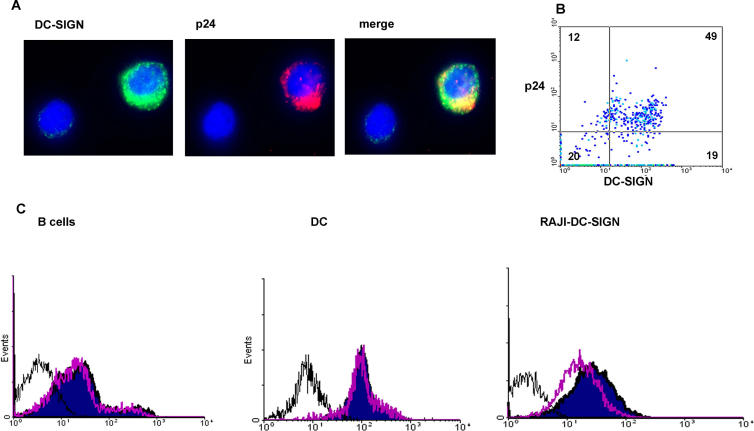
Expression of DC-SIGN and HIV-1 in B Cells (A) Immunofluorescent microscopy showing coexpression of DC-SIGN and HIV-1 in IL-4 and CD40L purified, activated B cells after 2 h of exposure to HIV-1 X4 (MN) at 37 °C. B cells not exposed to HIV-1 were used as controls; these included a subset that expressed DC-SIGN and were negative for HIV-1 (unpublished data). (600× magnification) Green indicates DC-SIGN, red indicates p24, and blue indicates DAPI. (B) Flow cytometry histograms showing coexpression of DC-SIGN and HIV-1 in IL-4 and CD40L purified, activated B cells after 2 h of exposure to HIV-1 X4 (MN) at 37 °C. (C) HIV-1 R5 (Ba-L) had no effect on expression of DC-SIGN by purified, activated B cells (left) or DC (middle), and minimally inhibited DC-SIGN expression in Raji–DC-SIGN cells (right) after 24 h incubation with the virus. Full histogram represents uninfected B cells; purple overlay histogram represents HIV-1–infected cells; and black overlay histogram represents isotype controls. Data are from single experiments representative of five independent experiments.

Based on the above results, we reasoned that DC-SIGN could be involved in B cell–mediated *trans* infection of T cells. To address this hypothesis, we first examined the relationship between DC-SIGN expression on purified B cells and uptake of HIV-1. Virus was expressed together with DC-SIGN in activated B cells after 2 h of exposure of the cells to virus as detected by immunofluorescent microscopy (Figure 5A). Quantitative immunofluorescence analysis by flow cytometry showed that 80% of B cells expressing HIV-1 p24 were DC-SIGN^+^ (Figure 5B). Our results also demonstrated that there was no alteration of DC-SIGN expression by B cells or DC loaded with HIV-1, although there was minimal down-regulation of DC-SIGN expression on HIV-1–infected, Raji–DC-SIGN cells by 24 h, using an MOI of 10^−4^ (unpublished data) or 10^−3^ (Figure 5C). This is in contrast to our recent findings that human herpesvirus 8 infects DC and macrophages via DC-SIGN and results in loss of surface expression of DC-SIGN [30]. Thus, after exposure of IL-4– and CD40L-activated B cells to HIV-1, the virus is associated with DC-SIGN^+^ but not DC-SIGN^−^ B cells, and there is no alteration in DC-SIGN expression in these cells as detected by flow cytometry.

**Figure 6:**
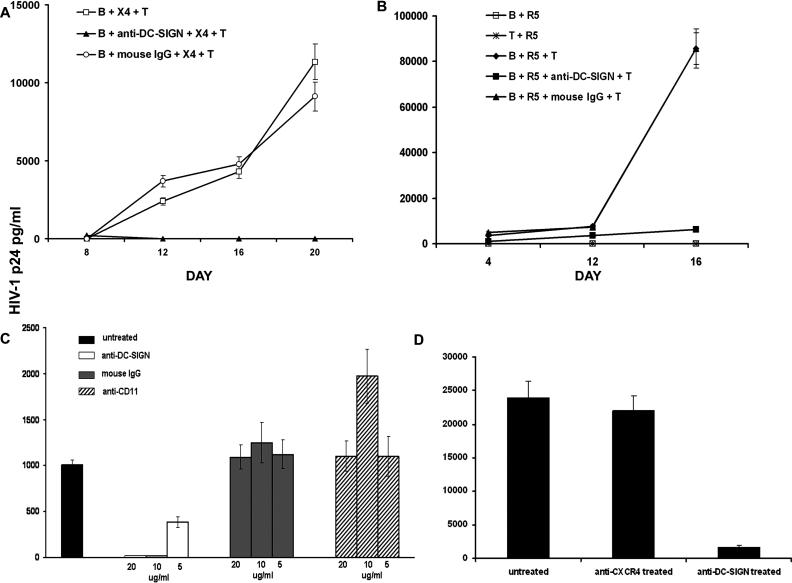
B Cell–Mediated Transmission of HIV X4 and R5 to T Cells Is Blocked by Anti–DC-SIGN mAb (A) and (B) HIV-1 p24 levels in cultures of activated B cells that were treated with anti–DC-SIGN mAb, incubated with either HIV-1 X4 (IIIb) (Figure 6A) or R5 (Ba-L) (Figure 6B) for 2 h at 37 °C, washed and co-cultured with autologous T cells. B and T cells cultured alone with each virus were used as controls. (C) HIV-1 p24 levels in cultures of activated B cells that were incubated with decreasing amounts of anti–DC-SIGN mAb prior to exposure to HIV-1 R5 (Ba-L) and culture with autologous T cells for 8 d. Treatment with mouse IgG or an unrelated mAb (anti-CD11a) had no effect on HIV-1 R5 (Ba-L) *trans* infection. (D) HIV-1 p24 levels in cultures of activated B cells incubated with anti–DC-SIGN mAb or anti-CXCR4 mAb, washed, loaded with HIV-1 X4 (IIIb), and co-cultured with autologous T cells for 12 d. Data are represented as mean of triplicates ± SE. Data are from single experiments representative of eight independent experiments.

We next determined the role of DC-SIGN on B cells in *trans* infection of T cells by treating IL-4– and CD40L-activated B cells with anti–DC-SIGN mAb or two nonspecific IgG as controls, one with the same isotype as the anti–DC-SIGN mAb (IgG2b) and the other of a different isotype (IgG1), prior to incubation with HIV-1 X4 or HIV-1 R5 and co-culture with T cells. The results show that pretreatment of B cells with anti–DC-SIGN mAb inhibited HIV-1 X4 and R5 replication in the B–T cell co-cultures, whereas pretreatment with the control IgG had no effect (Figures 6A and 6B). Also, inhibition of HIV-1 transmission by anti–DC-SIGN mAb was dose-dependent (Figure 6C). Because T cells do not express DC-SIGN [17], this virus-inhibitory effect was related to blocking of DC-SIGN on the activated B cells. These results indicate that HIV-1 X4 and R5 strains can be associated with B cells via DC-SIGN and that this leads to *trans* infection of T cells.

Although activated B cells did not express the primary CD4 receptor or the CCR5 coreceptor necessary for conventional infection by HIV-1 R5 (Figure 2B), they did express high levels of the CXCR4 coreceptor for HIV-1 (Figure 2B) [28]. Even though this is an inefficient receptor for HIV-1 infection in the absence of CD4 [31], we examined whether HIV-1 X4 *trans* infection of T cells was related to CXCR4 expression on the B cells. We treated activated B cells with anti-CXCR4 mAb or an IgG control, and showed that this did not inhibit HIV-1 X4 *trans* infection of T cells, whereas *trans* infection was blocked by pretreatment of the B cells with anti–DC-SIGN mAb (Figure 6D).

**Figure 7:**
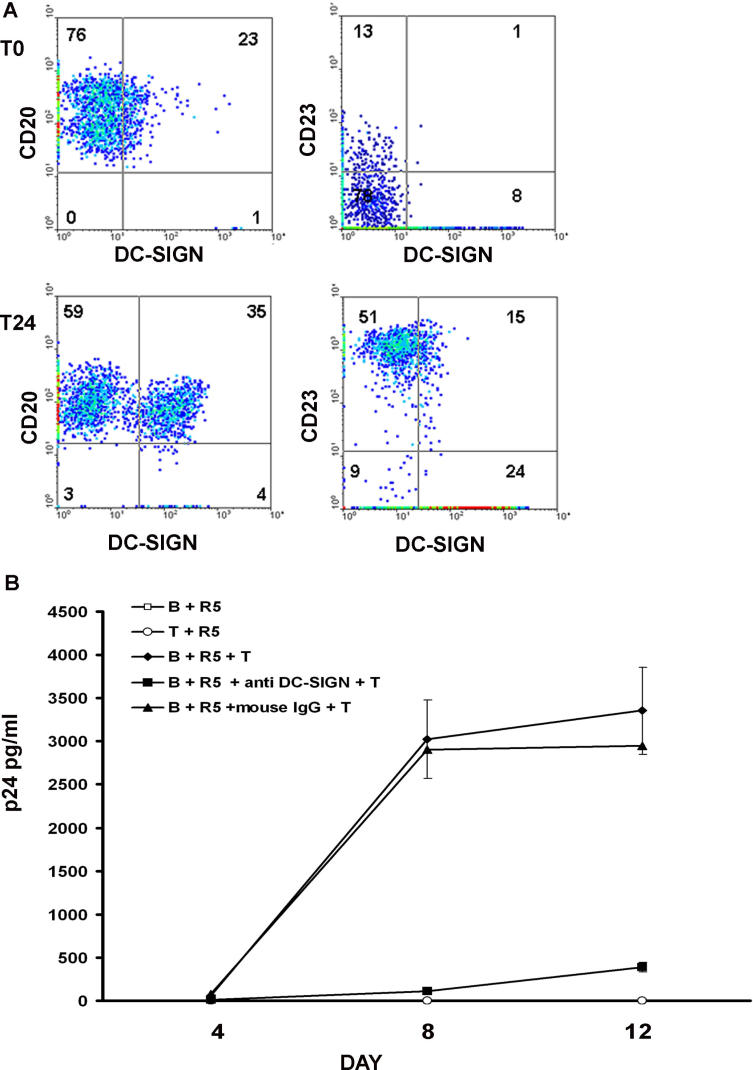
DC-SIGN–Expressing Tonsil B Cells Mediate *trans* Infection of T Cells (A) Expression of CD20 and DC-SIGN or CD23 and DC-SIGN in fresh (time 0 h; T0) and IL-4– and CD40L-activated (time 24 h; T24) B cells derived from tonsils. Data are from one experiment representative of five independent experiments. (B) Levels of HIV-1 p24 in co-cultures of B and T cells derived from tonsils. B cells were stimulated with IL-4 and CD40L for 24 h, treated with anti–DC-SIGN mAb or mouse Ig (20 μg/ml) or left untreated, and loaded with 10^−4^ MOI of HIV-1 R5 (Ba-L) for 2 h at 37 °C, then extensively washed in cold medium and incubated with purified, autologous CD4^+^ T cells (T). B cells and T cells directly loaded with HIV-1 served as controls. Amount of HIV-1 p24 in the cell culture supernatants was determined by ELISA. Data are from one experiment representative of two independent experiments.

Taken together, these results show that DC-SIGN expression on purified, IL-4– and CD40L-activated, peripheral blood B cells is directly related to *trans* infection of T cells. We hypothesized that this process could occur during B–T cell interactions in lymphatic tissues. In support of this, we found that DC-SIGN was expressed by 26.4 ± 6% (*n* = 5) of single-cell suspensions of purified tonsil B cells (representative example, T0, Figure 7A). In contrast to blood B cells, there was little coexpression of CD23 by freshly purified, tonsil B cells, but comparable up-regulation of CD23 and coexpression with DC-SIGN after 24-h stimulation with IL-4 and CD40L (T24, Figure 7A). Importantly, *trans* infection of T cells was mediated by the stimulated tonsil B cells, as demonstrated by an increase in p24 levels from <1 × 10^1^ pg per ml at day 4 to >3 × 10^3^ pg per ml at days 8 to12 in the B–T cell co-cultures (Figure 7B). Over 90% of this virus replication was blocked by pretreatment of the B cells with anti–DC-SIGN mAb. Thus, both DC-SIGN expressing blood and lymphatic tissue B cells can mediate efficient *trans* infection of T cells.

### HIV-1 Is Internalized in Activated B Cells

**Figure 8:**
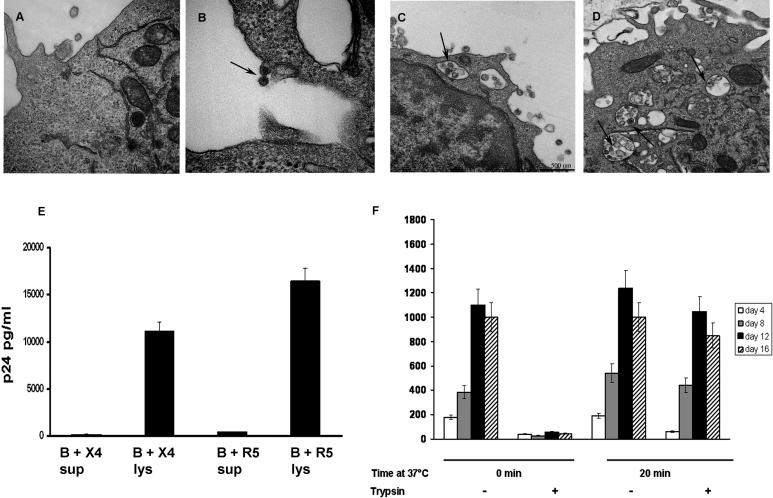
HIV-1 Is Internalized in B Cells (A–D) Transmission electron microscopy of HIV-1–loaded, activated B cells and DC. Activated B cells were left untreated (panel A) or loaded with AT-2–inactivated HIV-1 X4 (MN) for 1 h at 4 °C (panel B) or at 37 °C for 2 h (panel C). Immature DC (panel D) that were loaded with the same preparation of HIV-1 and incubated at 37 °C for 2 h were used as controls. Arrows indicate viral particles that have been internalized in cytoplasmic vesicles. (E) HIV-1 p24 levels in post-trypsin treatment supernatants (sup) and cell pellets of activated B cells (lys) that had been incubated with AT-2–inactivated HIV-1 X4 (MN) or HIV-1 R5 (Ba-L) for 2 h at 37 °C, and then incubated with 0.25% trypsin solution for 10 min at 37 °C. Controls included activated B cells without virus, which were negative for p24 (unpublished data). (F) Activated B cells were incubated with HIV-1 R5 (Ba-L) on wet ice, then washed and shifted at 37 °C for the times indicated. Cells were then treated with trypsin or mock treated and added to T cells. Supernatants were collected every 4 d and tested for p24. Data are from single experiments representative of three independent experiments.

HIV-1 is predominantly found within cytoplasmic vacuoles in DC after binding to DC-SIGN [3]. This could relate to different pathways of HIV-1 that are required for subsequent *trans* infection of T cells. We therefore conducted a series of experiments to assess whether HIV-1 was internalized by B cells. We first examined the physical association of HIV-1 with purified, activated B cells and DC by electron microscopy. As expected, no viral particles were observed in activated B cells not loaded with HIV-1 (Figure 8A). Activated B cells incubated with HIV-1 for 1 h at 4 °C had viral particles bound only to their outer surface (Figure 8B). However, activated B cells exposed to HIV-1 for 2 h at 37 °C had viral particles internalized in vacuoles of the cell cytoplasm (Figure 8C), with no intracellular virus apparent outside of vacuoles. Both intact viral particles and possible degraded virions were present in the vacuoles, similar to those observed after internalization of HIV-1 in DC (Figure 8D; [3]).

To prove further that HIV-1 particles were localized inside B lymphocytes and not merely bound to their surface, we incubated activated B cells with AT-2 inactivated HIV-1 X4 particles for 2 h at 37 °C to allow internalization, and then treated the cells with trypsin to cleave virions still bound on the cell surface. The culture medium was removed, and the cells were washed and lysed. HIV-1 p24 contained in the wash supernatants and cell lysates was measured by enzyme-linked immunosorbent assay (ELISA). Under these conditions, essentially all of the p24 was found in the whole cell lysate, as very little was detected in the cell culture supernatants collected after the trypsin treatment (Figure 8E). To prove that internalized virus and not surface-bound virus was transferred from B cells to the T cells, we next performed a protease protection transmission assay. Cells were loaded with HIV-1 R5 at 4 °C (wet ice) for 1 h, washed, and then shifted to 37 °C for the times indicated prior to treatment with trypsin or medium (mock treatment). As shown in Figure 8F, very brief proteolysis of the B cells that had been exposed to HIV-1 at 4 °C to inhibit virus entry and then shifted to 37 °C (i.e., 0 min incubation) prevented transmission of HIV-1 to the T cells. In contrast, proteolysis of the HIV-1–loaded B cells after shifting to 37 °C for 30 min did not prevent *trans* infection of the T cells. These data support that HIV-1 was internalized by the activated B cells prior to transmission to T cells.

**Figure 9:**
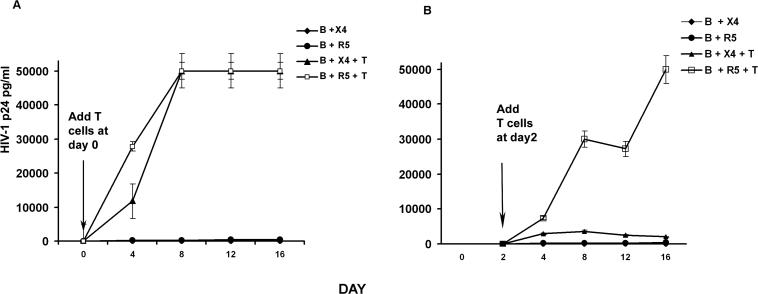
HIV-1 Is Maintained in an Infectious Form by B Cells Activated B cells were loaded with HIV-1 X4 (IIIb) or HIV-1 R5 (Ba-L) for 2 h at 37 °C, extensively washed and then either cultured immediately with autologous T cells (panel A) or incubated for 2 d prior to adding autologous T cells (panel B). B cells and T cells directly loaded with HIV-1 served as controls.

Finally, we examined the ability of HIV-1 to remain infectious in B cells over time, as has been shown for HIV-1 infection of DC [32]. For this, we exposed activated B cells to infectious X4 or R5 strains of HIV-1 for 2 h at 37 °C, and then either mixed them immediately with T cells or kept them at 37 °C for 2 d before mixing with T cells. As shown in Figure 9A and 9B, B cells that were loaded with HIV-1 and cultured alone for 2 d could still transmit virus to T cells, although at a lower efficiency then when T cells were added immediately to the HIV-1–exposed B cells. Taken together, these results indicate that HIV-1 was captured and internalized in activated B cells, where it remained infectious for T cells for at least 2 d.

## Discussion

Our results demonstrate that activated B lymphocytes derived from peripheral blood and lymphatic tissue express DC-SIGN, and that these cells mediate HIV-1 *trans* infection of T lymphocytes. Evidence for *trans* infection was an increase of HIV-1 p24 from undetectable levels (<1 × 10^1^ pg/ml) to >10^3^–10^4^ pg/ml after 12–16 d of co-culture of the activated B cells with autologous CD4^+^ T cells. The *trans* infection was not due to direct *cis* infection of the B cells, as virus replication was not detectable in B cells alone after exposure to HIV-1. Likewise, there was little or no detectable replication of HIV-1 after direct infection of T cells alone with the low input of HIV-1 used in our experiments. Expression of DC-SIGN by the B cells was associated with this enhanced HIV-1 infection of CD4^+^ T cells in *trans*. That is, treatment with IL-4 and CD40L, which are mediators of B cell activation by CD4^+^ T cells [19], enhanced DC-SIGN expression on the B cells. A total of 80% of the HIV-1–infected B cells were DC-SIGN^+^ by flow cytometry. This was supported by immunofluorescence microscopy analysis showing that DC-SIGN and HIV-1 were coexpressed in the B cell cytoplasm. Association of virus with a small portion of non-DC-SIGN–expressing B cells could be related to expression of other C-type lectin receptors by B cells that bind gp120 [16]. Most importantly, DC-SIGN expression by the B cells was required for *trans* infection of CD4^+^ T cells with both X4 and R5 tropic strains of HIV-1, as we could block essentially all of the *trans* infection by pretreating the B cells with mAb specific for DC-SIGN.

In further support of a central role for DC-SIGN on B cells in HIV-1 *trans* infection, we found that the number of DC-SIGN molecules expressed on activated B cells was similar to that known to be sufficient to sustain capture of HIV-1 and *trans* infection of T cells by DC [25]. Notably, the mechanism of *trans* infection of T cells by B cells did not involve HIV-1 infection of B cells by the conventional, CD4-CXCR4/CCR5 pathway. This was supported by the fact that B cells expressed little or no CD4 and did not express CCR5. Moreover, although B cells expressed high levels of CXCR4, treatment with anti-CXCR4 mAb prior to virus binding to the B cells did not inhibit *trans* infection of the T cells. Thus, although other C-type lectin receptors are expressed on B cells and can bind gp120 [16], our results support a requirement for DC-SIGN in B cell–mediated, *trans* infection of T cells.

HIV-1 was internalized by B cells as determined by resistance of B cell–associated virus to treatment with trypsin, and predominance of virus particles in cytoplasmic vacuoles of B cells after binding to DC-SIGN. Both intact and apparently degraded particles were present within the vacuoles, similar to HIV-1 internalization in DC via DC-SIGN [3]. Internalized and not extracellularly bound virus resulted in *trans* infection of T cells, which was demonstrated by lack of effect of trypsin treatment of HIV-1–loaded B cells on their ability to mediate *trans* infection. These results suggest a role for internalization of HIV-1 by B cells that is similar to that of DC in *trans* infection of T cells [33]. We further observed that virus was maintained for at least 2 d in an infectious form in B cells, comparable to its association with DC [32]. It is not clear how this virus infectivity persists. Recent studies indicate that most of the captured virions are destroyed in DC-SIGN–expressing DC and B cell lines engineered to express DC-SIGN [3,34]. However, a portion of the input virus can rapidly *trans* infect T cells [3,35]. In contrast, the longer term, persistent infectivity of DC-SIGN–expressing DC and B cell lines has been related to low levels of de novo HIV-1 replication [3,35]. It is possible that very low, subdetectable levels of HIV-1 replication occurred in our IL-4– and CD40L-stimulated B cells, and resulted in persistence of infectious virus involved in *trans* infection of T cells. Further work is in progress to delineate the mechanisms by which DC-SIGN–expressing B cells lead to HIV-1 *trans* infection of T cells.

The DC-SIGN–mediated, B-to-T cell *trans* infection pathway appears to be distinct from previously described B–T cell infectious processes. That is, it has been reported that transmission of HIV-1 from B cells to T cells involves virus trapped in immune complexes on the surface of the B cells [6–10]. This is not involved in our system, since in our studies, HIV-1 *trans* infection of T cells was mediated by B cells from normal, HIV-1 antibody–negative persons. Finally, in contrast to Swingler et al. [11], where *nef*-induced soluble factors released by infected macrophages work together with B cells to lead to *trans* infection of T cells, we observed *trans* infection of T cells by purified, DC-SIGN^+^ B cells in the absence of macrophages.

Notably, we found that approximately 8% of B cells in the blood of normal donors expressed DC-SIGN. Stimulation for 24 h with IL-4 and CD40L resulted in an increase in the number of B cells expressing DC-SIGN (i.e., 28%) and the level of DC-SIGN expression on the B cells. Approximately 80% of the DC-SIGN^+^ B cells in blood also expressed the type II C-type lectin receptor and B cell activation marker, CD23, which increased to 86% after stimulation in vitro with IL-4 and CD40L. Activation of these stimulated, DC-SIGN^+^ B cells was confirmed by elevated levels of T cell coreceptors CD58, CD80, and CD86, as well as increases in coexpression of the B cell signal transduction molecule CD22. Expression of DC-SIGN was not restricted to blood B cells, as we found that approximately 26% of tonsil B cells constitutively expressed DC-SIGN, and that this increased to 39% after stimulation of the cells with IL-4 and CD40L. Interestingly, our study showed that few B cells directly isolated from the tonsils expressed CD23, but that this number increased after stimulation with IL-4 and CD40L. Expression of CD23 on some but not all DC-SIGN^+^ B cells could be related to differential expression of CD23 by B cells in distinct areas of tonsils [36], and a pronounced cleavage and shedding of soluble CD23 by activated B cells [37].

Of interest is that there was a comparable number of DC-SIGN–expressing B cells in the blood of uninfected persons as in those with chronic HIV-1 infection who were treatment naive or receiving ART. In support of these findings, although B cells can harbor HIV-1 in blood [6], we found that HIV-1 did not kill B cells or inhibit DC-SIGN expression within 24–48 h of exposure to virus in vitro. Our observation that IL-4 and CD40L stimulation of B cells from persons receiving ART failed to enhance expression of DC-SIGN suggests a negative effect of ART on this process. This could be related to the reported inhibitory effect of protease inhibitors, which are common components of ART, on expression of DC-SIGN [38], and requires further study.

Expression of DC-SIGN on B cells suggests that it is operative in their normal function. This type II C-type lectin receptor could be involved in B cell trafficking and antigen presentation to T cells, similar to its function in DC [39]. Indeed, high levels of expression of DC-SIGN were induced in B cells by a combination of T cell factors IL-4 and CD40L and not by either alone. These results support the concept that DC-SIGN is involved in the interaction of activated B and T cells during antigen processing and presentation.

Our data indicate that DC-SIGN–expressing B cells could become vehicles for HIV-1 infection of T cells during their cognate interactions in the lymphatics. High concentrations of HIV-1 have been found associated with B cells and CD4^+^ T cells in lymph nodes [40], where B cells are activated and proliferate through cytokine and CD40L-CD40 interactions [41]. This has been related to virus-containing immune complexes on the surface of B cells that could lead to infection of CD4^+^ T cells during “cross talk” in the microenvironment of lymphoid tissues. The presence of a large amount of unspliced simian immunodeficiency virus RNA in B cell–rich areas of lymphoid tissues [42], is also consistent with virions being associated with B cells and follicular DC in germinal centers. B cells have therefore been proposed as an important, lymphatic reservoir in the pathogenesis of HIV-1 [28]. Our results suggest that B cells play a previously unrecognized role in replication of HIV-1 in T cells and viral pathogenesis through a DC-SIGN–dependent mechanism.

## Materials and Methods

### Donors.

PBMC were obtained from healthy, HIV-1–seronegative (*n* = 33) and HIV-1–seropositive (*n* = 20) adult volunteers and subjects enrolled in the Multicenter AIDS Cohort Study. Informed consent was obtained following institutional guidelines. ART-naive, HIV-1–seropositive subjects (*n* = 10) had mean (± SE) of 658 ± 124 CD4^+^ T cells per ml of blood, and HIV-1 RNA loads of 6,410 ± 3,701 copies per ml of plasma; HIV-1–seropositive subjects on ART (*n* = 10) had CD4^+^ T cell counts of 939 ± 82 and viral RNA loads of 66 ± 20. Human tonsils were obtained from patients (*n* = 5) undergoing therapeutic surgery, in accordance with institutional guidelines.

### Preparation of B and T cells.

PBMC were isolated by Ficoll-Hypaque density gradient separation and used immediately for surface phenotype staining or further purification. For B cell purification, monocytes were depleted with two rounds of anti-CD14 mAb-coated immunomagnetic microbeads (Miltenyi) according to the manufacturer's instructions. B cells (CD19^+^ cells) were then isolated from the CD14^−^ cell fraction by incubation with anti-CD19 mAb-coated, magnetic microbeads (Miltenyi Biotec, Bergisch Gladbach, Germany). The purity of the fractionated B cells was 96.4% ±0.4 (mean ± SE; *n* = 33) as determined by staining with anti-CD20 mAb, with <1% CD14^+^ and CD3^+^ cells. The immunomagnetic bead purification procedure did not alter expression of DC-SIGN or CD23 (unpublished data). Autologous CD4^+^ T cells were obtained from the remaining CD14^−^ CD19^−^ fraction by CD4^+^ cell purification using anti-CD4 mAb-coated microbeads (Miltenyi). Activated B cells were generated by culture of CD19^+^ cells in RPMI 1640 medium supplemented with 10% heat-inactivated FCS, 1,000 U/ml of rhIL-4 (R & D Systems, Minneapolis, Minnesota, United States), and 1 ug/ml of soluble trimeric CD40 L (Amgen, Thousand Oaks, California, United States). CD4^+^ T lymphocytes were cultured in RPMI1640 medium supplemented with 20% FCS and phytohemagglutinin (PHA) and IL-2, as described [43].

Tonsils were immediately transferred to the laboratory in cold PBS supplemented with penicillin (100 U/ml), streptomycin (100 μg/ml), gentamicin (5 μg/ml), ampothericin B (0.5 μg/m), and 5% FCS. The tissues were cut into fragments and pushed through a stainless steel sieve with a 250-μm mesh, using the flat end of a plastic syringe plunger, to remove the connective capsula. Lymphocytes from the collected cell suspension were isolated by Ficoll-Hypaque density gradient centrifugation. The cells collected at the interface were stained with anti-CD20, anti-CD3, and anti-CD14 mAb to determine the relative percentage of B and T lymphocytes, and were shown to contain 60%–70% B lymphocytes, 30%–40% T lymphocytes, and 1 % or less monocytes. B and T cells were purified by immunomagnetic bead separation and activated as described above.

### Preparation of DC.

DC were generated from CD14^+^ cells as described [44]. Briefly, CD14^+^ cells were enriched from PBMC with anti-CD14 mAb magnetic beads (>95% lineage negative, HLA DR^+^ cells) and cultured for 5 d in the presence of 1,000 U/ml of rhIL4 and rGM-CSF.

### Antibodies.

The following mAbs were used in this study: anti–DC-SIGN (CD209) (clone 120507; R & D Systems); anti-CD4, anti-CD14, anti-CD19, anti-CD20, anti-CD22, anti-CD45, anti-CD58, anti-CD80, anti-CD86, anti-CXCR4, and CCR5 (BD Pharmingen, San Diego, California, United States); anti-CD23 (Caltag Laboratories, Burlingame, California, United States); anti–HIV-1 p24 (KC57; Beckman-Coulter, Fullerton, California, United States). These mAbs were used either unlabeled or conjugated with FITC, PE, PE-Cy5, or PE-Cy7 as indicated below. Appropriate isotype-matched controls were used for background staining evaluation. For the virus receptor blocking experiments, we used sodium azide–free, low-endotoxin, purified, anti–DC-SIGN clone 120507 mAb and mouse IgG of the relevant isotype (Becton Dickinson, Palo Alto, California, United States) reconstituted in sterile PBS.

### Flow cytometry and quantification of DC-SIGN expression.

Expression of cell surface molecules was examined by flow cytometry with a Beckman Coulter XL flow cytometer. Cells were incubated with the desired antibodies or isotype controls for 30 min at 4 °C in buffer consisting of PBS supplemented with 0.1% FCS and 0.1% NaN_3_. After extensive washing with the buffer, the cells were resuspended in 1% paraformaldehyde in PBS for flow cytometric analysis. HIV-1 intracellular p24 was identify using PE-labeled KC57 anti–HIV-1 Gag mAb, following cell permeabilization using Permiflow (Invirion Diagnostics, Oak Brook, Illinois, United States) according to manufacturer's instructions. Results were expressed either as percent positive cells above the isotype control threshold or as MFI above the isotype controls.

For quantification of DC-SIGN expression on DC, Raji–DC-SIGN cells, and activated B cells, we used the Quantum Simply Cellular kit (Bangs Laboratories, Fishers, Indiana, United States) according to the manufacturer's instructions. Quantification was performed by converting the geometric mean channel fluorescence (GMCF) into ABC. The kit contains five microbeads of uniform size coated with different amounts of goat anti–mouse IgG (Fc-specific) on their surface that have different abilities to bind mouse antibodies (ranging from 0 to about 250,000 molecules). Both beads and cells were labeled with saturating amounts of FITC-conjugated, anti–DC-SIGN mAb, processed, and analyzed by flow cytometry under identical conditions. A calibrating curve was derived from the bead samples using QuickCal (Bangs Laboratories). The GMCF of the samples was converted to ABC per cell by comparison with the regression curve generated with the beads. Samples were also evaluated for fluorescence intensity as expressed by MESF units, using the Quantum MESF kit (Bangs Laboratories). The kit consists of five bead populations having different levels of FITC fluorescence intensity. As described above, a regression curve was generated by plotting the GMCF of the beads against their known MESF using QuickCal. The MESF of the cell samples was then determined as described above for ABC.

### Real-time RT-PCR measurement of DC-SIGN mRNA.

Total RNA was extracted from cells using Trizol (Invitrogen Life Sciences, Carlsbad, California, United States) according to the manufacturer's instructions, DNAse-treated (Ambion, Austin, Texas, United States) and affinity column purified (RNAeasy, Qiagen, Valencia, California, United States). The sequences of the primers and probe for Taqman amplification and detection of DC-SIGN mRNA were kindly provided by B. Lee (University of California, Los Angeles) and were DC-SIGN.F1, 5′-GCTGAGAGGCCTTGGATTCC-3′; DC-SIGN.R1, 5′-AGAGCGTGAAGGAGAGGAGTTG-3′; and DC-SIGN.probe, 5′-6FAM-ACCATGGCCAAGACACCCTGCTA -MGB-3′. The comparative threshold cycle (Ct) method [23,24] was used to determine relative mRNA expression levels. The primer and probe set for β-glucoronidase (β-GUS) (Applied Biosystems, Foster City, California, United States) was used as the endogenous control. Across all samples, mean β-GUS Ct values were 29 ± 1 (unpublished data). cDNA synthesis with 400-ng input RNA was performed in duplicate with Superscript II reverse transcriptase (Invitrogen) and random hexamers as described [24], in parallel with control reactions lacking RT. Amplification and detection for 40 cycles was performed on a Prism 7000 Sequence Detection System (Applied Biosystems). The fold-changes in DC-SIGN mRNA expression were calculated relative to appropriate calibrator samples, including untransfected K562 cells and unstimulated CD19^+^ B cells.

### Virus.

HIV-1 IIIb (X4 tropic virus) and HIV-Ba-L (R5 tropic virus) were propagated in PHA- and IL-2–activated, normal donor PBMC and purified as described [43]. Virus titers were determined as pg/ml by p24 ELISA (DuPont, Wilmington, Delaware, United States), with a lower limit of 1 × 10^1^ pg/ml and upper limit of ≥ 5 × 10^4^ pg/ml**.** AT-2–inactivated HIV-1 MN (CL.4/SUPT1; X4 tropic), and ADA (M/SUPT1-CCR5 CL.30; R5 tropic) were a gift from J. D. Lifson, National Cancer Institute, Frederick, Maryland.

### HIV-1 infection and transmission assay.

Purified, IL-4– and CD40L-stimulated B lymphocytes (1 × 10^6^) were incubated with different amounts of HIV-1 IIIb or HIV-1 Ba-L, i.e., 10^−3^, 10^−4^, or 10^−5^ MOI at 37 °C for 2 h. The MOI was based on tissue culture infectious dose 50% determined with susceptible CD8^−^, PHA-stimulated human lymphocytes and confirmed by spectrophotometric analysis of 10-fold serial dilutions on TZM-bl indicator cell line. Unless otherwise specified, cell-free supernatants were taken at various time intervals for titration of virus by p24 ELISA. No difference was observed in viability of mock-treated and HIV-1–treated B cells as measured by trypan blue dye exclusion. In some experiments, stimulated B cells were incubated with anti–DC-SIGN mAb (clone 120507) or CD11a/LFA-1 (clone HI111, BD Pharmingen) or mouse IgG (R & D Systems) for 30 min at 4 °C prior to incubation with virus. The specificity of anti–DC-SIGN mAb clone 120507 was confirmed by binding to DC-SIGN–transfected K-562 cells and lack of binding to K562 cells transfected with the DC-SIGN–related, type II C-type lectin, DC-SIGNR [30] .

### Loading of DC and activated B cells with HIV-1 for electron microscopy.

A total of 1 × 10^6^ DC or purified, activated B cells were incubated in a 1.5-ml Eppendorf tube with AT-2 HIV-1 (3 ng of p24 Ag /10^6^ cells) in a total volume of 100 μl at 37 °C for up to 2 h. After the incubation, cells were extensively washed in cold medium using a refrigerated microfuge, and the cell pellets were fixed in PBS with 2.5% glutaraldehyde for 1 h. Pellets were washed three times in PBS and then post-fixed in 1% aqueous osmium tetroxide supplemented with 1% K_3_Fe(CN)_6_ for 1 h. Pellets were then washed three times in PBS and then dehydrated through a series of 30%–100% ethanol, 100% propylene oxide, and then infiltrated with 1:1 mixture of propylene oxide–Polybed 812 epoxy resin (Polysciences, Warrington, Pennsylvania, United States) for 1 h. After several changes of 100% resin over 24 h, the pellet was embedded in a final change of resin, cured at 37 °C overnight, followed by additional hardening at 65 °C for two more days. Ultrathin (70 nm) sections were collected onto 200-mesh copper grids, stained with 2% uranyl acetate in 50% methanol for 10 min, followed by 1% lead citrate for 7 min. Sections were viewed using a JEM 1210 electron microscope (JEOL, Peabody, Massachusetts, United States).

### Immunofluorescence microscopy.

Purified, activated B cells loaded with AT-2 HIV-1 MN were spotted on poly-L-lysine–coated slides, fixed with 4% paraformaldehyde for 20 min, and then permeabilized with buffer (0.5% BSA, 0.1% saponin, 0.1%NaN_3_) for 20 min at room temperature. Cells were stained with FITC-conjugated, anti–DC-SIGN mAb and PE-labeled anti–HIV-1 p24 mAb. To avoid nonspecific binding of IgG, all incubations and dilutions of reagents were done in Super-Block blocking buffer (Pierce Biotechnology, Rockford, Illinois, United States). Controls included activated B cells not exposed to HIV-1.

## Supporting Information

Figure S1 CD40L and IL-4 Act Synergistically in Inducing Enhanced DC-SIGN and CD23 Expression on B Cells (A) Coexpression of DC-SIGN and CD23 on B cells from healthy, HIV-1–seronegative donors (*n* = 20). Hour 0 [T0] = 0 h; hour 24 [T24] = 24 h.
(B) Time-dependent expression of DC-SIGN and CD23 in response to IL-4 and CD40L. Purified B cells from a normal donor were cultured in the presence of IL-4 or CD40L or a combination of the two. Untreated cells were used as controls (NT). DC-SIGN and CD23 coexpression on activated B cells was greatest using a combination of 1,000 U/ml of IL4 and 1 μg/ml of CD40L for 24 h. Single concentrations of 100, 500, and 10,000 U/ml of IL-4 and 0.1, 0.5, and 10 μg/ml of CD40L induced similar, low levels of DC-SIGN expression (unpublished data). Also, various combinations of these concentrations of IL-4 and CD40L induced less DC-SIGN expression than this combination (unpublished data).
(380 KB JPG)

### Accession Numbers

The GenBank accession number (http://www.ncbi.nlm.nih.gov/Genbank) for the gene mentioned in this paper is *CD209* (NM_021155).

## Abbreviations

ABC: antibody binding capacity
ART: antiretroviral therapy
CD40L: CD40 ligand
DC: dendritic cell
DC-SIGN: DC-specific ICAM-3 grabbing nonintegrin
ELISA: enzyme-linked immunosorbent assay
IL: interleukin
mAb: monoclonal antibody
MESF: molecules of equivalent soluble fluorochrome
MFI: mean fluorescence intensity
MOI: multiplicity of infection
PBMC: peripheral blood mononuclear cells
SE: standard error

## References

[ppat-0020070-b001] Geijtenbeek TB, Kwon DS, Torensma R, van Vliet SJ, van Duijnhoven GC (2000). DC-SIGN, a dendritic cell-specific HIV-1-binding protein that enhances trans-infection of T cells. Cell.

[ppat-0020070-b002] Geijtenbeek TB, van Kooyk Y (2003). Pathogens target DC-SIGN to influence their fate. DC-SIGN functions as a pathogen receptor with broad specificity. APMIS.

[ppat-0020070-b003] Turville SG, Santos JJ, Frank I, Cameron PU, Wilkinson J (2004). Immunodeficiency virus uptake, turnover, and 2-phase transfer in human dendritic cells. Blood.

[ppat-0020070-b004] De Milito A (2004). B lymphocyte dysfunctions in HIV infection. Curr HIV Res.

[ppat-0020070-b005] Clapham PR, McKnight A (2002). Cell surface receptors, virus entry and tropism of primate lentiviruses. J Gen Virol.

[ppat-0020070-b006] Moir S, Malaspina A, Li Y, Chun TW, Lowe T (2000). B cells of HIV-1-infected patients bind virions through CD21-complement interactions and transmit infectious virus to activated T cells. J Exp Med.

[ppat-0020070-b007] Malaspina A, Moir S, Nickle DC, Donoghue ET, Ogwaro KM (2002). Human immunodeficiency virus type 1 bound to B cells: relationship to virus replicating in CD4+ T cells and circulating in plasma. J Virol.

[ppat-0020070-b008] Jakubik JJ, Saifuddin M, Takefman DM, Spear GT (2000). Immune complexes containing human immunodeficiency virus type 1 primary isolates bind to lymphoid tissue B lymphocytes and are infectious for T lymphocytes. J Virol.

[ppat-0020070-b009] Dopper S, Wilflingseder D, Prodinger WM, Stiegler G, Speth C (2003). Mechanism(s) promoting HIV-1 infection of primary unstimulated T lymphocytes in autologous B cell/T cell co-cultures. Eur J Immunol.

[ppat-0020070-b010] Jakubik JJ, Saifuddin M, Takefman DM, Spear GT (1999). B lymphocytes in lymph nodes and peripheral blood are important for binding immune complexes containing HIV-1. Immunology.

[ppat-0020070-b011] Swingler S, Brichacek B, Jacque JM, Ulich C, Zhou J (2003). HIV-1 Nef intersects the macrophage CD40L signalling pathway to promote resting-cell infection. Nature.

[ppat-0020070-b012] McKay PF, Imami N, Johns M, Taylor-Fishwick DA, Sedibane LM (1998). The gp200-MR6 molecule which is functionally associated with the IL-4 receptor modulates B cell phenotype and is a novel member of the human macrophage mannose receptor family. Eur J Immunol.

[ppat-0020070-b013] Ryan EJ, Marshall AJ, Magaletti D, Floyd H, Draves KE (2002). Dendritic cell-associated lectin-1: A novel dendritic cell-associated, C-type lectin-like molecule enhances T cell secretion of IL-4. J Immunol.

[ppat-0020070-b014] Bates EE, Fournier N, Garcia E, Valladeau J, Durand I (1999). APCs express DCIR, a novel C-type lectin surface receptor containing an immunoreceptor tyrosine-based inhibitory motif. J Immunol.

[ppat-0020070-b015] Bonnefoy JY, Lecoanet-Henchoz S, Aubry JP, Gauchat JF, Graber P (1995). CD23 and B-cell activation. Curr Opin Immunol.

[ppat-0020070-b016] He B, Qiao X, Klasse PJ, Chiu A, Chadburn A (2006). HIV-1 envelope triggers polyclonal Ig class switch recombination through a CD40-independent mechanism involving BAFF and C-type lectin receptors. J Immunol.

[ppat-0020070-b017] Geijtenbeek TB, Torensma R, van Vliet SJ, van Duijnhoven GC, Adema GJ (2000). Identification of DC-SIGN, a novel dendritic cell-specific ICAM-3 receptor that supports primary immune responses. Cell.

[ppat-0020070-b018] Banchereau J, de Paoli P, Valle A, Garcia E, Rousset F (1991). Long-term human B cell lines dependent on interleukin-4 and antibody to CD40. Science.

[ppat-0020070-b019] Lederman S, Cleary AM, Yellin MJ, Frank DM, Karpusas M (1996). The central role of the CD40-ligand and CD40 pathway in T-lymphocyte-mediated differentiation of B lymphocytes. Curr Opin Hematol.

[ppat-0020070-b020] Relloso M, Puig-Kroger A, Pello OM, Rodriguez-Fernandez JL, de la Rosa G (2002). DC-SIGN (CD209) expression is IL-4 dependent and is negatively regulated by IFN, TGF-beta, and anti-inflammatory agents. J Immunol.

[ppat-0020070-b021] Satomi M, Shimizu M, Shinya E, Watari E, Owaki A (2005). Transmission of macrophage-tropic HIV-1 by breast-milk macrophages via DC-SIGN. J Infect Dis.

[ppat-0020070-b022] Puig-Kroger A, Serrano-Gomez D, Caparros E, Dominguez-Soto A, Relloso M (2004). Regulated expression of the pathogen receptor dendritic cell-specific intercellular adhesion molecule 3 (ICAM-3)-grabbing nonintegrin in THP-1 human leukemic cells, monocytes, and macrophages. J Biol Chem.

[ppat-0020070-b023] Perkin-Elmer (1997). Relative quantitation of gene expression: ABI Prism 7700 Sequence Detection System. User Bulletin 2. Revision B.

[ppat-0020070-b024] Godfrey TE, Kim SH, Chavira M, Ruff DW, Warren RS (2000). Quantitative mRNA expression analysis from formalin-fixed, paraffin-embedded tissues using 5′ nuclease quantitative reverse transcription-polymerase chain reaction. J Mol Diagn.

[ppat-0020070-b025] Pohlmann S, Baribaud F, Lee B, Leslie GJ, Sanchez MD (2001). DC-SIGN interactions with human immunodeficiency virus type 1 and 2 and simian immunodeficiency virus. J Virol.

[ppat-0020070-b026] Geijtenbeek TB, van Kooyk Y (2003). DC-SIGN: A novel HIV receptor on DCs that mediates HIV-1 transmission. Curr Top Microbiol Immunol.

[ppat-0020070-b027] Engering A, Van Vliet SJ, Geijtenbeek TB, Van Kooyk Y (2002). Subset of DC-SIGN(+) dendritic cells in human blood transmits HIV-1 to T lymphocytes. Blood.

[ppat-0020070-b028] Moir S, Lapointe R, Malaspina A, Ostrowski M, Cole CE (1999). CD40-mediated induction of CD4 and CXCR4 on B lymphocytes correlates with restricted susceptibility to human immunodeficiency virus type 1 infection: Potential role of B lymphocytes as a viral reservoir. J Virol.

[ppat-0020070-b029] Castro BA, Weiss CD, Wiviott LD, Levy JA (1988). Optimal conditions for recovery of the human immunodeficiency virus from peripheral blood mononuclear cells. J Clin Microbiol.

[ppat-0020070-b030] Rappocciolo G, Jenkins FJ, Hensler HR, Piazza P, Jais M (2006). DC-SIGN is a receptor for human herpesvirus 8 on dendritic cells and macrophages. J Immunol.

[ppat-0020070-b031] Philpott SM (2003). HIV-1 coreceptor usage, transmission, and disease progression. Curr HIV Res.

[ppat-0020070-b032] Trumpfheller C, Park CG, Finke J, Steinman RM, Granelli-Piperno A (2003). Cell type-dependent retention and transmission of HIV-1 by DC-SIGN. Int Immunol.

[ppat-0020070-b033] Kwon DS, Gregorio G, Bitton N, Hendrickson WA, Littman DR (2002). DC-SIGN-mediated internalization of HIV is required for trans-enhancement of T cell infection. Immunity.

[ppat-0020070-b034] Moris A, Nobile C, Buseyne F, Porrot F, Abastado JP (2004). DC-SIGN promotes exogenous MHC-I-restricted HIV-1 antigen presentation. Blood.

[ppat-0020070-b035] Nobile C, Petit C, Moris A, Skrabal K, Abastado JP (2005). Covert human immunodeficiency virus replication in dendritic cells and in DC-SIGN-expressing cells promotes long-term transmission to lymphocytes. J Virol.

[ppat-0020070-b036] Dono M, Zupo S, Colombo M, Massara R, Gaidano G (2003). The human marginal zone B cell. Ann N Y Acad Sci.

[ppat-0020070-b037] Bonnefoy JY, Lecoanet-Henchoz S, Gauchat JF, Graber P, Aubry JP (1997). Structure and functions of CD23. Int Rev Immunol.

[ppat-0020070-b038] Whelan KT, Lin CL, Cella M, McMichael AJ, Austyn JM (2003). The HIV protease inhibitor indinavir reduces immature dendritic cell transendothelial migration. Eur J Immunol.

[ppat-0020070-b039] Geijtenbeek TB, Krooshoop DJ, Bleijs DA, van Vliet SJ, van Duijnhoven GC (2000). DC-SIGN-ICAM-2 interaction mediates dendritic cell trafficking. Nat Immunol.

[ppat-0020070-b040] Pantaleo G, Graziosi C, Demarest JF, Cohen OJ, Vaccarezza M (1994). Role of lymphoid organs in the pathogenesis of human immunodeficiency virus (HIV) infection. Immunol Rev.

[ppat-0020070-b041] Grewal IS, Flavell RA (1998). CD40 and CD154 in cell-mediated immunity. Annu Rev Immunol.

[ppat-0020070-b042] Reinhart TA, Rogan MJ, Viglianti GA, Rausch DM, Eiden LE (1997). A new approach to investigating the relationship between productive infection and cytopathicity in vivo. Nat Med.

[ppat-0020070-b043] Balachandran R, Thampatty P, Enrico A, Rinaldo C, Gupta P (1991). Human immunodeficiency virus isolates from asymptomatic homosexual men and from AIDS patients have distinct biologic and genetic properties. Virology.

[ppat-0020070-b044] Wang QJ, Huang XL, Rappocciolo G, Jenkins FJ, Hildebrand WH (2002). Identification of an HLA A*0201-restricted CD8(+) T-cell epitope for the glycoprotein B homolog of human herpesvirus 8. Blood.

