# Correction: DC-SIGN on B Lymphocytes Is Required for Transmission of HIV-1 to T Lymphocytes

**DOI:** 10.1371/journal.ppat.0020088

**Published:** 2006-08-25

**Authors:** Giovanna Rappocciolo, Paolo Piazza, Craig L Fuller, Todd A Reinhart, Simon C Watkins, David T Rowe, Mariel Jais, Phalguni Gupta, Charles R Rinaldo

In *PLoS Pathogens*, volume 2, issue 7: DOI: 10.1371/journal.ppat.0020070

Page 3, column 1, line 3 should have included this reference for the Raji-DC-SIGN cells:

Wu L, Martin TD, Carrington M, KewalRamani VN (2004) Raji B cells, misidentified as THP-1 cells, stimulate DC-SIGN-mediated HIV transmission. Virology 318: 17-23.

The Acknowledgments should have included a thank you to V. N. KewalRamani (National Cancer Institute) for the Raji-DC-SIGN cells.

